# Implementing a Parallel Image Edge Detection Algorithm Based on the Otsu-Canny Operator on the Hadoop Platform

**DOI:** 10.1155/2018/3598284

**Published:** 2018-05-13

**Authors:** Jianfang Cao, Lichao Chen, Min Wang, Yun Tian

**Affiliations:** ^1^Department of Computer Science and Technology, Xinzhou Teachers University, Xinzhou 034000, China; ^2^College of Computer Science and Technology, Taiyuan University of Science and Technology, Taiyuan 030024, China

## Abstract

The Canny operator is widely used to detect edges in images. However, as the size of the image dataset increases, the edge detection performance of the Canny operator decreases and its runtime becomes excessive. To improve the runtime and edge detection performance of the Canny operator, in this paper, we propose a parallel design and implementation for an Otsu-optimized Canny operator using a MapReduce parallel programming model that runs on the Hadoop platform. The Otsu algorithm is used to optimize the Canny operator's dual threshold and improve the edge detection performance, while the MapReduce parallel programming model facilitates parallel processing for the Canny operator to solve the processing speed and communication cost problems that occur when the Canny edge detection algorithm is applied to big data. For the experiments, we constructed datasets of different scales from the Pascal VOC2012 image database. The proposed parallel Otsu-Canny edge detection algorithm performs better than other traditional edge detection algorithms. The parallel approach reduced the running time by approximately 67.2% on a Hadoop cluster architecture consisting of 5 nodes with a dataset of 60,000 images. Overall, our approach system speeds up the system by approximately 3.4 times when processing large-scale datasets, which demonstrates the obvious superiority of our method. The proposed algorithm in this study demonstrates both better edge detection performance and improved time performance.

## 1. Introduction

Edges are a basic image feature and they are present between a target and a background and between two targets, two regions, or two primitives. Most of an image's information is carried in the edges. Usually, an image edge is a set of pixels around which the gray-level values exhibit a step change. Thus, edge detection technology is based on the discontinuity or mutation of gray levels or textural characteristics between an object and its background. Edge detection is an important aspect of image processing and is the basis of many analytical methods in such fields as image segmentation, pattern recognition, machine vision, and regional shape extraction. In addition, edge detection is an important step in image analysis and 3D reconstruction and is therefore also an important feature in the field of digital image analysis. Image edge detection algorithms have been widely studied [1]. The basic idea is as follows. First, an edge enhancement operator is used to highlight local edges in an image. Then, the edge strength of the image is defined, and the edge points are extracted by setting a threshold [2]. The performance of the edge detection algorithm directly affects the precision of extracted object contours and the performance of the system. In 1959, Julesz [3] was the first to discuss edge detection; later, in 1965, Roberts [4] began to systematically study edge detection. After nearly 60 years of research, many different edge detection methods have been designed, and each has its own characteristics and limitations.

With the advent of the big data era, traditional edge detection technologies are facing problem of poor edge detection and long runtimes; thus, this technology needs to be further analyzed and studied. The Canny operator is no exception. Tang and Long [5] proposed a fast implementation of the Canny operator based on a GPU+CPU combination in which the GPU was used to parallelize the Canny edge detection algorithm. Their results showed that the processing time for 8-bit images at a resolution of 1024 × 1024 was 122 ms, as well as a speedup ratio of approximately 5.39 times compared to the traditional Canny edge detection algorithm. Such architectural improvements can improve the performance of traditional image edge detection algorithms under single-node architectures when processing massive datasets. However, GPU-based parallel designs involve high hardware costs and require developers who clearly understand the target computer hardware systems; furthermore, such implementations have difficulties due to the high cost of communications among nodes [6].

In recent years, the MapReduce framework on the Hadoop platform has attracted extensive attention from scholars and researchers. MapReduce is a parallel computing model that targets a distributed environment. It provides developers with a complete programming interface that makes a deep understanding of the underlying computer system unnecessary. Furthermore, it is both less expensive and more functional than traditional computing models. Due to these advantages, MapReduce has become a hotspot in the field of parallel design [7]. Therefore, this study aims to apply the MapReduce programming model on the Hadoop platform to parallelize the Canny operator so that it better meets the needs of big data processing.

## 2. Related Works

Classic image edge detection algorithms include both first-order differential operators (i.e., the Roberts, Prewitt, Sobel, and Canny operators) and second-order differential operators (i.e., the Laplacian and LoG operators) and can be applied to a wide range of applications. Wang and Liu applied the Roberts operator to detect vehicle image edges and recognize vehicle license plate locations by combining it with mathematical morphology [8]. Yang et al. used the Roberts operator and linear fitting to obtain two boundaries and proposed an Otsu thresholding image segmentation method [9]. To calculate image quality more consistently compared to subjective evaluations, Zhang et al. designed a new image quality assessment algorithm that used the Prewitt operator to extract vertical edges in the HSV color space [10]. Dwivedi et al. presented a handwritten Sanskrit word-recognition method using the Prewitt operator for character edge detection [11]. Nguyen et al. built an architecture for the real-time hardware cosimulation of edge detection using the Prewitt edge operator to evaluate the real-time performance of an edge detection algorithm [12]. Singh et al. applied the Sobel operator to detect edges in real-time surveillance videos and presented a new resource-efficient FPGA-based hardware architecture [13]. Jiang et al. performed color image segmentation using the Canny operator by combining it with a pulse-coupled neural network [14]. Based on the color vector angle and the Canny operator, Tang et al. proposed a robust image hashing mechanism to achieve a desirable tradeoff of classification performances between rotation robustness and discrimination [15]. Sun and Han enhanced images by extracting the edge and texture information from the original images using the Laplacian operator [16]. For glass fragment images, Yang et al. proposed an edge detection method that combined the LoG operator and mathematical morphology and established a rapid transmission system to realize rapid edge detection [17]. Amer and Abushaala performed a comparative study on typical classical edge detection operators [18]. By comparing experiments from existing studies, the following conclusions were drawn. Although the abovementioned detection algorithms have the advantages of being simple and easy to implement and provide good real-time performance, they also have obvious shortcomings. The image edge feature extracted by the Roberts operator is relatively rough and provides inaccurate edge locations. The edge features extracted by the Prewitt operator have wide margins and many discontinuities. Similarly, the Sobel operator does not provide accurate locations of image edges. The Laplacian operator is highly sensitive to noise, and the LoG operator cannot eliminate salt-and-pepper noise in an image. In contrast, the Canny operator applies an optimization principle to detect image edges and sets the high and low thresholds using the gradient histogram of the image, which gives edge detection a high signal-to-noise ratio and improves its reliability. The Canny operator can obtain satisfactory edge detection results when there is only one image background and when the gray-level changes between the background and target are not too large [19]. However, in reality, images are easily affected by factors such as the environment and illumination, which can cause substantial gray-level changes between the image background and a target, which degrades the edge detection performance of the traditional Canny operator because the parameters must be adjusted manually. Such weak contrast conditions reduce the adaptability of the Canny algorithm, causing it to easily fail to detect edges [20]. Various researchers have improved the traditional Canny operator and proposed some improved Canny edge detection algorithms based on adaptive thresholds. Ronggui et al. presented an automatic road extraction method for vague aerial images using an improved Canny edge detection operator that included automatic thresholding to segment the image into a binary edge image [21]. Guiming and Jidong realized remote sensing image edge detection based on an improved Canny operator [22]. However, with the arrival of the big data era, traditional algorithms have become insufficient when faced with massive images. The algorithms' computational load increases dramatically, and their runtime performance declines sharply. Thus, extracting edge features from large-scale digital image collections faces new challenges.

Hadoop is an open-source software framework developed by the community at large and distributed under the Apache License; programmers can use it to develop distributed programs without knowing the underlying details of the framework [23]. The core design of the Hadoop framework includes the Hadoop distributed file system (HDFS) and MapReduce. HDFS provides distributed storage for large amounts of data, and MapReduce implements distributed parallel computing, which provides a new approach for improving the edge detection performance on massive images. O'Driscoll et al. discussed how to apply big data technologies, such as the Apache Hadoop project, to process and analyze petabyte- (PB-) scale datasets within the bioinformatics community [24]. Lee et al. conducted a survey on MapReduce to assist the database and open-source communities in understanding various technical aspects of the MapReduce parallel programming framework [25]. MapReduce is widely used in various fields. Alham et al. applied the MapReduce framework to the SVM algorithm and proposed a MapReduce-based distributed SVM algorithm (MRSMO) for automatic image annotation [26]. Cao et al. designed a parallel particle swarm optimization- (PSO-) optimized BP neural network algorithm using the MapReduce framework to realize massive scene image classification [27]. Li et al. used MapReduce to parallelize the fast fuzzy c-means algorithm to achieve large-scale underwater image segmentation [28]. Cao et al. parallelized the traditional *k*-means algorithm in a MapReduce environment to retrieve large-scale scene images [29]. The above studies all improved the runtime performance and system efficiency of various algorithms using the MapReduce parallel programming model, and the number of MapReduce-based applications is gradually increasing. However, there are few studies concerning the Otsu algorithm or that investigate the MapReduce distributed parallel processing of edge detection operators and their application to the field of digital image processing.

To solve the abovementioned problems, this study proposes a parallel image edge detection algorithm based on the Otsu operator by optimizing the thresholds of the Canny operator on the Hadoop platform. The proposed method improves the Canny edge detection algorithm in the OpenCV function library using the Otsu algorithm. Then, the MapReduce parallel programming model on the Hadoop platform is applied to realize the parallel Otsu-Canny edge detection algorithm in a cluster environment and achieve parallel processing for the feature extraction task for massive numbers of images. Compared with the single-node architecture, the proposed approach reduces the running time of the proposed algorithm by approximately 67.2% when using a Hadoop cluster architecture consisting of 5 nodes and an image scale of 60,000 images. The system achieves a speedup of approximately 3.4 times, which reflects its obvious superiority in processing large-scale datasets. Our approach significantly reduces the computational load and improves the runtime and edge detection performance for images, greatly increasing the speed of edge feature extraction through task decomposition.

## 3. Otsu-Canny Edge Detection Algorithm

The Canny edge detection operator is a multilevel edge detection algorithm developed by John F. Canny in 1986; this operator uses a method called calculus of variations to find a function that optimizes a particular function. The goal is to find an optimal edge detection algorithm that retains the original image attributes [30].

### 3.1. Canny Algorithm Principle

The traditional Canny operator detects image edges by performing the following steps sequentially.

*(1) Applying Gaussian Smoothing to Images Using Gaussian Convolution. *To eliminate image noise, the Canny operator takes the first derivative of the two-dimensional Gaussian function (formula (1)) as a noise filter; then, it performs convolution processing to smooth the image:

(1) $$\begin{array}{c} G\left(\;x,y\;\right)=\frac{1}{2\pi \sigma^{2}}\exp\left[\;-\frac{x^{2}+y^{2}}{2\sigma^{2}}\;\right], \end{array}$$

where *σ* represents the standard deviation of the Gaussian filter function, which is manually set and controls the image smoothness.

*(2) Filtering the Image Using the First Derivative of the Gaussian Operator to Obtain the Gradient Intensity and Direction of the Image. *Suppose that *I*(*i*, *j*) is a smooth image. The first derivative of the image in the *x* and *y* directions is

(2) Pxi,j=12Ii,j+1−Ii,j+Ii+1,j+1−Ii+1,j,Pyi,j=12Ii,j−Ii+1,j+Ii,j+1−Ii+1,j+1.

Thus, the gradient intensity *M*[*i*, *j*] and gradient direction *θ*[*i*, *j*] are

(3) Mi,j=Pxi,j2+Pyi,j2,

(4) θi,j=arctanPyi,jPxi,j,

respectively.

*(3) Performing Nonmaximum Suppression Along the Gradient Direction*. Divide the gradient directions into 8 directions: 0°–180°, 45°–22.5°,90°–270°, and 135°–31.5°. Compare the value of the derivative of each pixel with the modulus of adjacent pixels in the image for these 8 directions along the edge detection points of the argument direction. Finally, take the pixel with the maximum partial derivative as an edge point.

*(4) Detecting and Connecting the Edge Points Using the Dual-Threshold Method. *The traditional Canny operator requires manually setting the high threshold *T*_high_ and low threshold *T*_low_, the relation of which is generally *T*_low_ = 0.5*T*_high_. When the gradient intensity *M*(*i*, *j*) of a pixel (*i*, *j*) is greater than the high threshold value *T*_high_, the point is marked as an edge point, and when the gradient intensity *M*(*i*, *j*) of the pixel (*i*, *j*) is less than the low threshold value *T*_low_, the pixel cannot be an edge point. When the gradient intensity of the pixel is between *T*_high_ and *T*_low_, the point is marked as a candidate edge point; further judgments are later made by combining each candidate point with its surrounding pixels.

### 3.2. Otsu-Optimized Threshold Values of the Canny Operator

The Otsu operator, also called the maximum class square error method, is a self-adapting threshold determination method that can solve the Canny operator's problem, which is that it is unable to select the high and low thresholds adaptively according to the image characteristics [31]. The Otsu operator uses the thresholds to divide the image into two parts: the background and the target. The best threshold occurs when the difference between the two parts is the largest (i.e., the maximum variance between classes is achieved). The method for determining the optimal threshold value for the Otsu operator is as follows.

Suppose that *t* is the segmentation threshold between the background and target of image *I*(*i*, *j*), the grayscale range *G* of the image is [0, *L* − 1], and the probability of each grayscale is *p*_*i*_. The threshold *t* divides the image into two categories: *G*_0_ = [0, *t*] and *G*_1_ = [*t* + 1, *L* − 1]. Then, the following are true.

The probabilities of the two classes *G*_0_ and *G*_1_ are *α*_0_ = ∑_*i*=0_^*t*^*p*_*i*_ and *α*_1_ = 1 − *α*_0_, respectively.

The average gray values of the two classes *G*_0_ and *G*_1_ are *μ*_0_ = *μ*_*t*_/*α*_0_ and *μ*_1_ = (*μ* − *μ*_*t*_)/(1 − *α*_0_), respectively, where *μ* = ∑_*i*=0_^*L*−1^*i* × *p*_*i*_ and *μ*_*t*_ = ∑_*i*=0_^*t*^*i* × *p*_*i*_.

The between-cluster variance of the two classes *G*_0_ and *G*_1_ is

(5) $$\begin{array}{c} \eta^{2}\left(\;t\;\right)=\alpha_{0}{\left(\;\mu_{0}-\mu \;\right)}^{2}+\alpha_{1}{\left(\;\mu_{1}-\mu \;\right)}^{2}=\alpha_{0}\alpha_{1}{\left(\;\mu_{0}-\mu_{1}\;\right)}^{2}. \end{array}$$

Then, the high threshold *T*_high_ of the Canny operator can be obtained by solving max(*η*^2^(*t*)).

## 4. Parallel Implementation of the Otsu-Canny Edge Detection Algorithm

Recently, big data has attracted increasing attention. Big data often possess multiple sources, complex semantics, and large scales, and they are heterogeneous, dynamic, and changeable, which brings new challenges to traditional machine learning technologies. Traditional machine learning methods focus on data analysis with an appropriate statistical method from datasets with relatively small numbers of samples to find the function and value of the data. However, one of the core objectives of big data technologies is to extract the potential rules from huge amounts of data with extremely complex structures to maximize the value of the data. Therefore, both the runtime performance and the system efficiency of traditional algorithms decrease sharply when applied to big data.

Although the Otsu-Canny algorithm improves the performance of the traditional Canny edge detection algorithm, when it is applied to larger datasets, the time it requires for edge detection increases as well, eventually raising efficiency issues. The MapReduce parallel programming framework in the Hadoop platform provides a distributed parallel computing environment for big data processing. Faced with the rapid growth in data in the big data era, there is a need to improve the time efficiency and edge detection performance of the Canny algorithm. Therefore, this study intends to address the low time efficiency problem and the poor edge detection performance of the traditional Canny algorithm optimized by the Otsu algorithm as proposed in the literature [18]. To this end, this study implemented a parallel version of the Otsu-Canny edge detection algorithm using the MapReduce parallel programming framework.

### 4.1. Hadoop Platform and the MapReduce Programming Model

Hadoop is a software framework for the distributed processing of large-scale data, and it is a widely accepted big data platform. Hadoop implements a distributed file system called HDFS, which provides high fault tolerance, can be deployed on inexpensive hardware, provides high throughput for accessing application data, and is suitable for applications involving large datasets [32].

**Figure 1:**
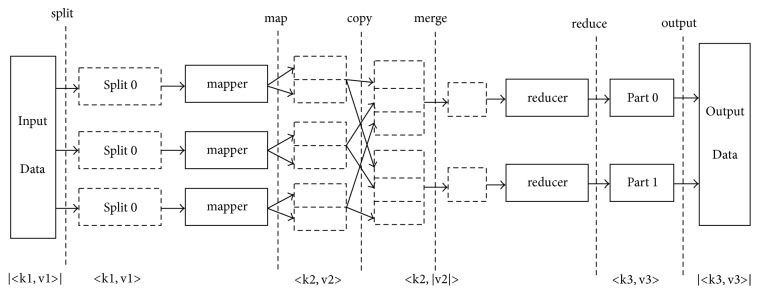
The MapReduce programming model process.

As one of the core subprojects of Hadoop, MapReduce is a parallel programming model that distributes computing tasks and data to Hadoop cluster nodes, allowing all nodes to perform tasks in parallel to obtain intermediate results, then subsequently summarizing the intermediate results and distributing additional computing tasks to each node to obtain the final results [33]. When performing these tasks, MapReduce divides calculations into two tasks, Map and Reduce, with the help of a functional programming method. The input and output of each task take the form of key-value pairs, and the mapper() and reducer() functions are designed to achieve the mapping from one key-value pair to another key-value pair. A flowchart of the MapReduce process is shown in Figure 1.

### 4.2. Parallel Design and Implementation of the Otsu-Canny Algorithm

#### 4.2.1. General Framework for Parallel Image Edge Detection

**Figure 2:**
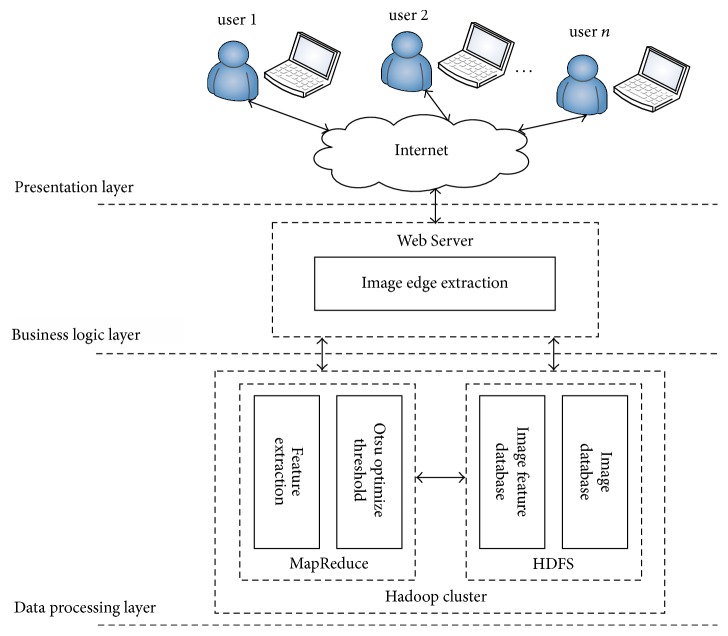
Architecture for massive image edge extraction.

The general architecture of the parallel Otsu-Canny edge detection on the Hadoop platform is shown in Figure 2.

The general architecture is divided into three layers.

1. Presentation layer: users access services through the Internet through which they can submit images or receive edge detection results.
2. Business logic layer: a web server executes the corresponding processing tasks according to the users' requests.
3. Data processing layer: this layer is the core of the entire system architecture and it is mainly responsible for storage, management, optimization of the threshold for the Canny operator, edge feature extraction, and outputting the results for massive numbers of images. Users submit their images to the Hadoop distributed system, which then performs threshold optimization and edge feature extraction and outputs the results.

The specific processes are as follows. First, the images in the massive image database are processed into the input format* SequenceFile* of a Hadoop job. Then, the Map task segments the image files into splits in accordance with the default slice size (128 MB) of the Hadoop system, each of which may contain multiple image files. Next, the MapReduce framework is used to optimize the threshold and extract the edge features of the image in a parallel manner using key-value pairs such as <image name, image file>. Finally, key-value pairs (such as <image name, image edge feature>) are generated and written to the HDFS distributed file system of the Hadoop platform.

#### 4.2.2. Design and Realization of the Algorithm

**Figure 3:**
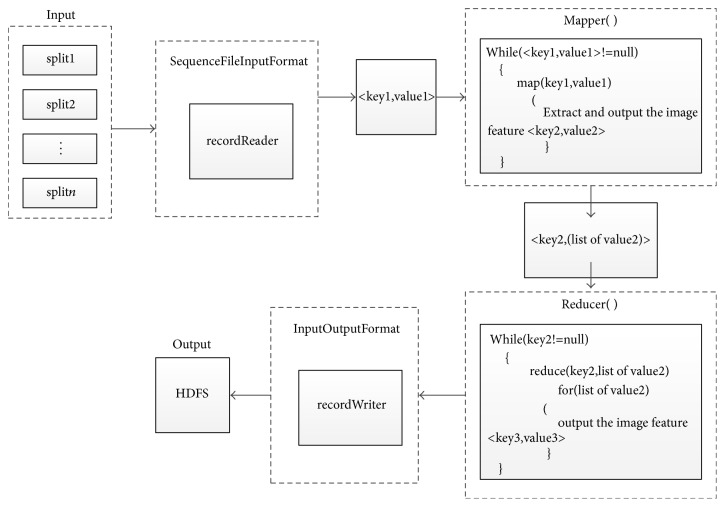
The workflow of the mapper() and reducer() functions.

Because OpenCV is an open-source and cross-platform computer vision library that has been used to realize many algorithms for image processing and computer vision and provides a large number of Java interfaces [34], this study improved the Canny operator using the OpenCV function library and implemented the parallel image edge detection algorithm based on the Otsu-Canny operator using Java on the Hadoop platform using a total of approximately 600 lines of source code. The most important Map and Reduce tasks of the proposed algorithm are provided as complete source code implementations in the Supplementary Materials (available here). The workflow of the mapper() and reducer() functions is shown in Figure 3. MapReduce transforms splits into key-value pairs (<key1, value1>) using the recordReader method of SequenceFileInputFormat, in which key1 is the path name of the image and value1 is a pointer to the image data. The mapper() function returns another key-value pair (<key2, value2>). The MapReduce functionality merges all the values that have the same key to generate <key2, (value2 list)>, which serves as the input to the reducer() functions. After reducer() processing the output key-value pair (<key3, value3>) is written to the HDFS file system by the RecordWriter functions in the ImageOutputFormat custom category.

*Definition of Image Data Type.* Because the Hadoop framework does not define the class associated with the image as the data type of the key-value pair* <key, value>* and because the Hadoop framework specifies that a user-defined data type can only be used by implementing the* Writable* interface, this study defines the data type* RawImage* and rewrites the basic input and output method defined by the* Writable* interface in Hadoop. Unlike other data types, the* RawImage* data type exposes some new functions to implement reading and storing images, such as converting images to the* Mat* type of a single channel or three channels and encoding the* Mat* type into an image file. These functions facilitate combining Hadoop with OpenCV.

*Design of Input/Output Format for Jobs.* The image pixel information would be destroyed if the image were to be split to store and process it in a distributed manner on the Hadoop platform. Therefore, we adopt the whole image as the value of the key-value pair.

We define the input format class of the image file. Then, we use the input format* SequenceFileInputFormat*, which is a built-in Hadoop format that takes the* SequenceFile* file as input. The* SequenceFileInputFormat* format segments the* SequenceFile* file into splits and sends it to the Map task. Each split contains multiple records, and each record is an image (image name as* key*, image content as* value*), which is a good solution to the problem of starting too many Map tasks due to too many small files.

We also defined the output format class of the image file. The class* FileOutputFormat* describes the output format of the data. We designed the class* ImageOutputFormat*, which inherits from* FileOutputFormat* and is used to write the <key, value> provided by the users to a file with a specified format. The* ImageRecordWriter* class inherits from the class* RecordWriter<Text, RawImage>*, which regards the image name as the key and the instance of the* RawImage* type as the value to be stored in the HDFS file system.

*Design and Realization of mapper() Function. *The main mapper() tasks include reading images, processing images, and converting data. The main mapper() code is as follows.

    mapper (Text Key, RawImage value)
    { 
     Mat img = value.toMat (); //Convert value to
    * Mat* matrix
  Imgproc.cvtColor (img, img_Gray, Imgproc. COLOR_BGR2GRAY);
            //Transform the image into
             the gray image
  Thresh = Imgproc.threshold (img_Gray, dst, 0, 255,
  Imgproc.THRESH_BINARY+Imgproc. THRESH_OTSU);
     //Use Otsu threshold segmentation method to
     obtain the high threshold of the image *T*_high_
     Imgproc. Canny (img_Gray, Cannyop, *T*_high_*∗*0.5, *T*_high_);
    //Take *T*_high_*∗*0.5 as the low threshold and
    extract the Canny edge feature of the image
    mos.write (fileName, RawImage.toImage
     (Cannyop), fileName.toString());
             // Write file to HDFS file
             system using multioutput
             mode
     }

*Design and Realization of reducer() Function. *The main reducer() tasks include reduction and consolidation, sorting, and outputting the results. The main reducer code is as follows.

  reducer(Text Key, RawImage value)
 
{ 
    for(RawImage value: values)
    { 
      mos.sort (key, value, key.toString()); //Reduction and sorting
      mos.write (key, value, key.toString());
          // Write file to HDFS file system
          using multioutput mode
     }
 
 }

*Result Output of Image Edge Detection.* The default output file name of Hadoop takes the form* name-r(m)-nnnnn*, where* name* is set by the user, *r* represents the output of the Reduce task, *m* represents the output of the Map task, and *nnnnn* is an integer indicating the block number. However, to facilitate subsequent processing and display images, we rewrote the* getDefaultWorkFile()* method in the* FileOutputFormat *class using the following code, which causes the output filename to be in the form “filename.jpg.”

  public Path getDefaultWorkFile (TaskAttemptContext context,
  String extension) throws IOException
    { 
     FileOutputCommitter committer =
      (FileOutputCommitter) getOutputCommitter
    (context);
return new Path (committer.getWorkPath(), getUniqueFile (context, getOutputName (context), extension));
     } //Get a task submission

In this study, we use the Hadoop multifile output format* MultipleOutputs* and write the output to the file system in the form of records, in which the image file name is regarded as the key of the key-value pair, and the image file is regarded as the value of the key-value pair. The main code is as follows.

  ImageRecordWriter extends RecordWriter <Text, RawImage>
 
{ 
   write (Text fileName, RawImage img)
   { 
     FSDataOutputStream out = fs.create
      (outputPath);
     out.write (img.getRawData ());
    } // Write the image data to the output path, using the specified file name
 
 }

## 5. Experiment and Result Analysis

To validate the performance of the parallel Otsu-Canny edge detection algorithm proposed in this study, we tested it on an image edge feature extraction task for a large number of images on the Hadoop platform.

### 5.1. Experimental Environment and Data

The experimental environment was a Hadoop cluster composed of five computers (one master node and four slave nodes) configured in an intranet. All the nodes were equipped with Intel Core 4.2 GHz quad-core, 8-thread processors, 8 GB memory, 4 TB hard disks, JDK1.7.0_79, and the 64-bit Ubuntu 14.04 operating system, and Hadoop-2.5.1 (64-bit compiled version) was used.

The experimental data stemmed from Pascal VOC2012 [32], which consists of 17,125 images, involving people, vehicles, animals and plants, indoor and outdoor scenes, and other categories (20 categories total), and is freely available to researchers. To verify the performance of the proposed algorithm when addressing massive image datasets, we constructed a massive image database by copying images from the Pascal VOC2012 dataset.

### 5.2. Experimental Results and Analysis

To validate the performance of the proposed algorithm in this study, we conducted experimental comparisons by evaluating aspects such as the edge detection performance, running time, system speedup, scaleup, and sizeup.

#### 5.2.1. Image Edge Detection Performance

**Figure 4:**
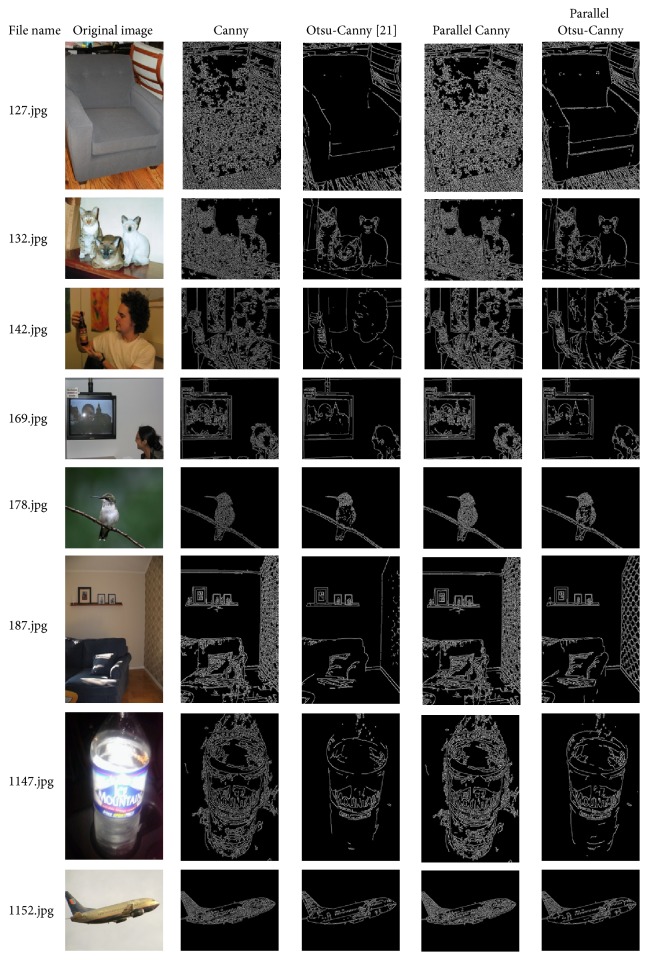
Comparison of the edge detection performance of different algorithms.

Using the Pascal VOC2012 image database, the traditional serial Canny algorithm, the Otsu-Canny algorithm in the literature [21], the parallel Canny algorithm, and the parallel Otsu-Canny algorithm proposed in this study were compared in terms of their edge detection performances. The experimental results are shown in Figure 4.

As shown in Figure 4, using different edge detection algorithms, the edge detection performance of the algorithm proposed in this study is preferable to the traditional Canny algorithm, the Otsu-Canny algorithm, and the parallel Canny algorithm; it not only effectively retains the texture information of the original image but also includes few false edges and results in better-connected edges. Furthermore, because the Otsu algorithm in the Hadoop cluster architecture finds better thresholds, the algorithm proposed in this paper is more accurate in terms of image edge location and better at processing image details, which indicates that edge detection performance improvements do not occur by chance.

#### 5.2.2. Running Time.

**Figure 5:**
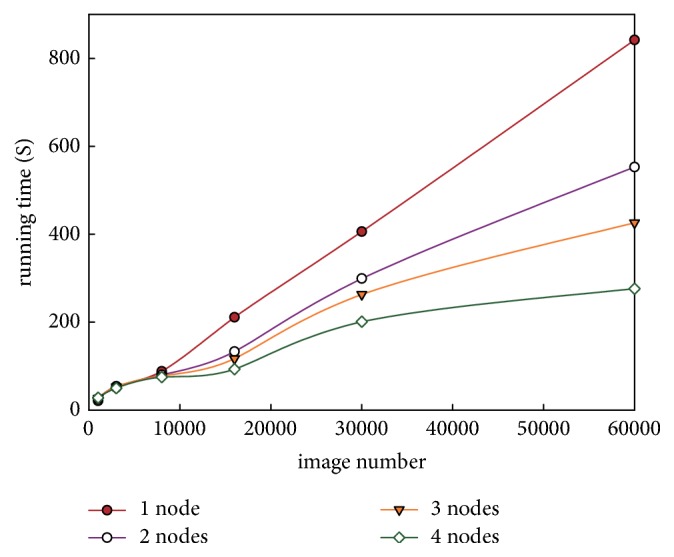
Comparison of the running times on the Hadoop cluster nodes.

To further verify the effectiveness of the proposed approach, we constructed datasets of different sizes by replication.  Table 1 and Figure 5 report a comparison of the edge detection time for the different edge detection approaches and different numbers of Hadoop cluster nodes while varying the number of images.

The data in Table 1 show the running times required for different numbers of images. The running times required by the Canny and Otsu-Canny algorithm in the literature [21] are much longer than those of the parallel Canny algorithm and the parallel algorithm proposed in this study and become dramatically longer as the number of images increases because the parallel algorithm adopts the distributed parallel processing technology of the MapReduce framework in the Hadoop platform, whereas the Canny and Otsu-Canny algorithm in the literature [18] use a single-node architecture with limited processing capacity. Because the complexities of the Canny algorithm and Otsu-Canny algorithm when executing in parallel are similar, the running times of the parallel Canny algorithm and the algorithm proposed in this study are nearly identical under the MapReduce programming model. However, the edge detection effect of the parallel Canny algorithm is similar to that of the traditional Canny algorithm as shown in Figure 4. Therefore, the proposed algorithm in this study achieves better edge detection while simultaneously improving the running time, combining the experimental results shown in Figures 4 and 5.

Figure 5 shows a comparison of the running times for the different Hadoop cluster nodes. As shown in Figure 5, when the number of images is below 10,000, the running time reduction for image edge detection is not very obvious when multiple nodes are used. Instead, the running time of the multinode cluster architecture is slightly longer than that of the single-node architecture when processing small-scale image datasets due to the increased communication overhead among node computers. However, with a sharp increase in the image scale, the advantage of the multinode architecture of the Hadoop cluster gradually becomes apparent. Although the running time with different numbers of Hadoop cluster nodes increases as the number of images increases, the time consumption of the single-node architecture grows approximately linearly, whereas that of the multinode architecture increases more slowly; in addition, using a larger number of computer nodes results in a gentler running time curve. These results fully demonstrate the superiority of the Hadoop cluster architecture when processing big data. The running time is reduced by approximately 67.2% using a Hadoop cluster architecture with 5 nodes when the number of images reaches 60,000. It is obvious from Table 1 and Figure 5 that increasing the number of images has little influence on the time performance of the parallel algorithm based on the MapReduce framework, further illustrating the advantages of distributed parallel processing.

#### 5.2.3. Speedup, Sizeup, and Scaleup

Typically, for the parallel programming model based on MapReduce on the Hadoop platform, we adopt three important indicators, speedup, sizeup, and scaleup [35], to evaluate the computational performance of the proposed algorithm in this study. For these experiments, we still randomly selected and replicated images from the Pascal VOC2012 image database to construct different datasets.

**Figure 6:**
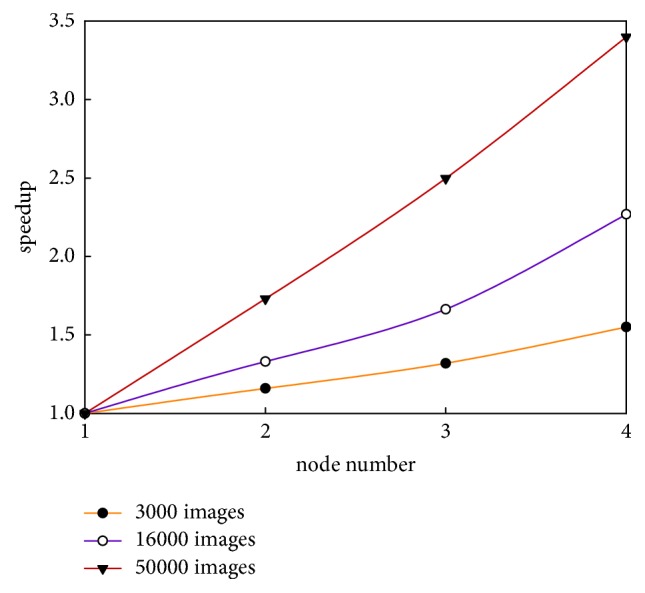
Comparison of speedup.

Speedup [36] refers to the ratio of the time consumed to run a task under the single-node architecture to the time consumed to run the same task under the multinode architecture. Theoretically, the speedup should increase linearly. However, speedup does not grow linearly due to communication costs and load balancing, among other factors.  Figure 6 shows the experimental comparisons of the speedup of the approach proposed in this study using datasets of different scales.

In Figure 6, the speedup presents the speedup growth trends as the number of nodes in the Hadoop cluster increases; increased data size leads to an increased speedup magnitude. For the same dataset, the computing speed of the system improves as the number of nodes in the Hadoop cluster increases—that is, the computing time decreases. Therefore, the speedup curve takes on a growth trend. With regard to the different datasets, a larger number of images result in better performance by the multinode architecture, and the computing speed becomes much higher compared to that of the single-node architecture. Furthermore, the system speedup is almost linear when the number of images reaches 50,000, which clearly demonstrates the superior performance of the MapReduce parallel processing on the Hadoop platform.

**Figure 7:**
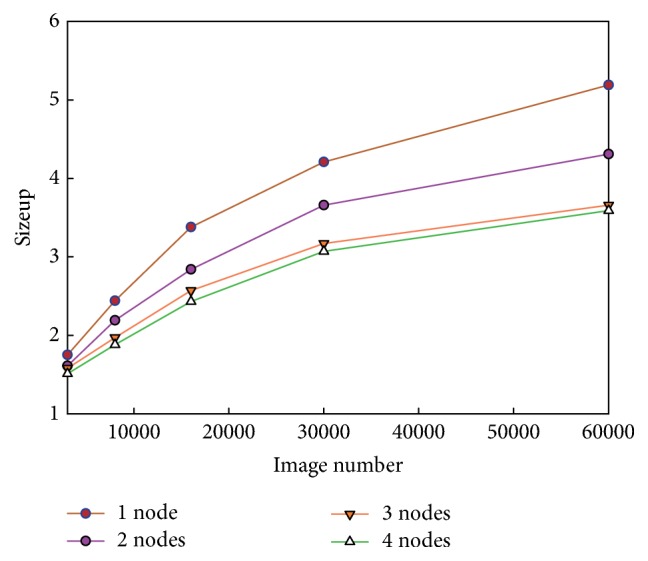
Comparison of sizeup.

Sizeup is defined as how much longer a task takes on a given system when the data scale is *m*-times larger than the original data scale. In other words, as the data size increases, a higher sizeup means that the Hadoop cluster will take longer to complete the task. To evaluate the sizeup, we varied the number of slave nodes in the Hadoop cluster from 1 to 4 and varied the number of images from 3,000 to 60,000. The experimental results are shown in Figure 7.

As Figure 7 shows, when the image scale increases from 3,000 to 60,000, the sizeup increases by 2.97 for the 1-node cluster, whereas it increases by only 2.34 for the 4-node system. The sizeup for the 4-node cluster grows less due to increased communication time among the nodes. Although the communication time of the 4-node cluster system is longer than that of the 1-node system, the communication time does not increase significantly under the proposed approach in the study as the number of images increases. Therefore, the approach proposed in this study obtains a good sizeup performance.

**Figure 8:**
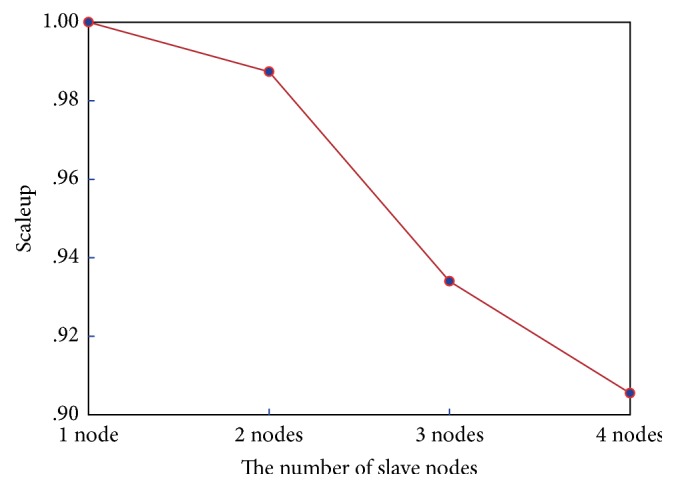
Comparison of scaleup.

Scaleup is often used to measure the performance of an algorithm when increasing the system and data sizes, and it refers to the capability of an *m*-times larger system to complete an *m*-times larger job within the same running time as the original system. Therefore, the scaleup value illustrates how much better an algorithm addresses big data when more slave node computers are available in the Hadoop cluster. Specifically, a higher scaleup value denotes a better algorithm performance. To measure scaleup, we obtain different scaleup values by increasing the number of slave nodes and the image scale simultaneously using the following combinations: (1-node cluster, 3,000 images), (2-node cluster, 5,000 images), (3-node cluster, 30,000 images), and (4-node cluster, 60,000 images).  Figure 8 shows the experimental results.

We can see that the scaleup values in Figure 8 are all above 0.90, which confirms the better performance of the algorithm proposed in this study.

## 6. Conclusions

Edge detection is a basic problem in the image processing field. The accuracy of edge detection strongly influences subsequent operations such as feature extraction, object recognition, 3D reconstruction, image matching, and quantitative analysis. The rapid development of network technology and multimedia technology has resulted in a sharp increase in the number of available images—far exceeding the abilities of traditional image processing algorithms. The algorithms for extracting edge features from images are no exceptions. Currently, the open-source Hadoop platform is widely used because it is convenient and inexpensive for building clustered systems. Moreover, it provides an easy-to-use parallel distributed storage and programming model. Researchers in both academia and industry are developing new ways to apply algorithms developed for the traditional single-node architecture environment in Hadoop cluster environments.

This study presented a parallel Otsu-Canny edge detection algorithm based on the MapReduce framework of the Hadoop platform to realize edge feature extraction from massive numbers of images. Moreover, it fostered a deeper exploration and discussion of the parallel design and realization of the Otsu-Canny algorithm. This study investigated three topics: combining the MapReduce parallel programming model with a traditional edge detection algorithm, the parallel design and implementation of the Otsu-Canny algorithm, and fast and effective automated edge feature extraction for massive numbers of images on the Hadoop platform. The completed algorithm was tested with images from the Pascal VOC2012 image database. The experimental results indicate that the proposed algorithm can not only address massive datasets but also achieve good system performance in terms of the speedup, sizeup, and scaleup evaluation metrics. The experimental results also demonstrate that the proposed algorithm can take full advantage of the resources of the distributed cluster system to improve the algorithm's edge detection performance and that its parallelization is very good. In addition, the distributed parallel cluster system based on the MapReduce framework on the Hadoop platform improved the runtime performance of the algorithm significantly compared to traditional algorithms with a single-node architecture, which demonstrates the strong computing ability of distributed parallel processing.

The analysis and processing of big data, especially image data, have become a hot research topic with the arrival of the big data era. In the future, we plan to conduct a study that considers the following aspects: optimizing the design of the Map and Reduce tasks used in the MapReduce framework and real-time large-scale image edge detection processing.

## Figures and Tables

**Table 1:** Comparison of the running times of different algorithms under different data scales.

Image scale	Running time (S)			
	Canny algorithm	Otsu-Canny algorithm [21]	Parallel Canny algorithm	The proposed approach (4 slave nodes)
1,000	30	30	27	28
3,000	58	60	51	50
8,000	92	95	76	75
16,000	155	159	93	93
30,000	487	488	199	201
60,000	840	843	275	276

## Data Availability

This work involved data from the Pascal VOC2012 image database, which is publicly accessible at http://cvlab.postech.ac.kr/~mooyeol/pascal_voc_2012/ [32].

## Supplementary Materials

Supplementary Materials This section consists of the core source codes of the Map and Reduce tasks, respectively.
