# Supplementary material for: Implementing a Parallel Image Edge Detection Algorithm Based on the Otsu-Canny Operator on the Hadoop Platform
Source: Comput Intell Neurosci. 2018 May 13;2018:3598284. doi: 10.1155/2018/3598284 (PMC5971336; doi:10.1155/2018/3598284)
Supplement: Supplementary Materials — This section consists of the core source codes of the Map and Reduce tasks, respectively. [file 3598284.f1.pdf]

Attachment:

The core source code of the edge detection of the Otsu-Canny operator in the Hadoop platform (ImageCanny.java)

```
//Map task is as follows.

package bishe;

import java.io.IOException;

import org.apache.hadoop.fs.Path;

import org.apache.hadoop.io.Text;

import org.apache.hadoop.mapreduce.Job;

import org.apache.hadoop.mapreduce.Mapper;

import org.apache.hadoop.mapreduce.Mapper.Context;

import org.apache.hadoop.mapreduce.lib.input.FileInputFormat;

import org.apache.hadoop.mapreduce.lib.input.SequenceFileInputFormat;

import org.apache.hadoop.mapreduce.lib.output.FileOutputFormat;

import org.apache.hadoop.mapreduce.lib.output.LazyOutputFormat;

import org.apache.hadoop.mapreduce.lib.output.MultipleOutputs;

import org.opencv.core.Core;

import org.opencv.core.CvType;

import org.opencv.core.Mat;

import org.opencv.imgproc.Imgproc;

import bishe.Image.RawImage;

public class ImageCanny
```

```
{public static class CannyMapper extends Mapper<Text, RawImage, Text, RawImage>
{
    private MultipleOutputs<Text,RawImage> mos;
    protected void setup(Context context) throws IOException, InterruptedException
    {
        super.setup(context);
        mos=new MultipleOutputs<Text,RawImage>(context);
                                          // Create multiple file output objects
    }
    @Override
    protected void map(Text key, RawImage value, Context context)
        throws IOException, InterruptedException
        {
            // TODO Auto-generated method stub
            Path path = new Path(key.toString());
            Text fileName = new Text(path.getName());
            Mat img = value.toMat();
             // Parse the original image file directly into the Mat type with three channels
            Imgproc.cvtColor(img, img, Imgproc.COLOR_BGR2GRAY);
            Mat Cannyop=new Mat();
            Mat dst=Mat.zeros(img.rows(), img.cols(), CvType.CV_8UC(3));
```

```
            double thresh=  Imgproc.threshold(img, dst, 0, 255,
                            Imgproc.THRESH_BINARY+Imgproc.THRESH_OTSU);
                              // Get the adaptive threshold using the Otsu algorithm
            Imgproc.Canny(img, Cannyop, 0.5*thresh, thresh);
        // Obtain the edge detection image of the Canny operator and stored it in the Cannyop
            mos.write(fileName, RawImage.toImage(Cannyop),fileName.toString());
                                                // Output results to the HDFS
      }
      protected void cleanup(Context context) throws IOException, InterruptedException
      {
         super.cleanup(context);
          mos.close();
       }
}
public static void main(String[] args) throws IOException, ClassNotFoundException,
InterruptedException
{
      Job job = Job.getInstance();
      job.setJobName("ImageCanny");
      job.setJar("ImageCannyTest.jar");
      job.setJarByClass(ImageCanny.class);
```

```
        job.addFileToClassPath(new Path("hdfs://master:9000/opencv-300.jar"));
        job.addFileToClassPath(new Path("hdfs://master:9000/libopencv_java300.so"));
        FileInputFormat.addInputPath(job, new Path("hdfs://master:9000/ImageSeq"));
        FileOutputFormat.setOutputPath(job, new Path("hdfs://master:9000/output"));
        job.setInputFormatClass(SequenceFileInputFormat.class);
        LazyOutputFormat.setOutputFormatClass(job, ImageOutputFormat.class);
        job.setMapperClass(CannyMapper.class);
        job.setOutputKeyClass(Text.class);
        job.setOutputValueClass(RawImage.class);
        job.waitForCompletion(true);
    }
}

//Reduce task is as follows.
package bishe;
import java.io.IOException;
import org.apache.hadoop.fs.Path;
import org.apache.hadoop.io.Text;
import org.apache.hadoop.mapreduce.Reducer;
import org.apache.hadoop.mapreduce.lib.output.MultipleOutputs;
import bishe.Image.RawImage;
public class Image_Readuce_Canny extends Reducer <Text, RawImage, Text, RawImage>
{
```

```
    private MultipleOutputs<Text,RawImage> mos;
    protected void setup(Context context) throws IOException, InterruptedException
    {
        super.setup(context);
        mos=new MultipleOutputs<Text,RawImage>(context);
     }
     protected void reduce(Text key, Iterable<RawImage> values, Context context
                  ) throws IOException, InterruptedException
    {
        for(RawImage value: values)
            mos.sort(key,value,key.toString( ));
            mos.write(key,value,key.toString());
     }
}
```
